# Mitigating Drought Stress in Maize: Synergistic Effects of Zinc Sulfate and *Pseudomonas* spp. on Physiological and Biochemical Responses

**DOI:** 10.3390/plants14101483

**Published:** 2025-05-15

**Authors:** Fahimeh Khaledi, Hamidreza Balouchi, Mohsen Movahhedi Dehnavi, Amin Salehi, Beata Dedicova

**Affiliations:** 1 Department of Agronomy and Plant Breeding, Faculty of Agriculture, Yasouj University, Yasouj 7591874831, Iran; fahime.khaledi1990@yahoo.com (F.K.); movahhedi1354@yu.ac.ir (M.M.D.); aminsalehi@yu.ac.ir (A.S.); 2 Department of Plant Breeding, Swedish University of Agricultural Sciences (SLU), Alnarp, 234 22 Lomma, Sweden

**Keywords:** antioxidant enzymes, chlorophyll, plant-growth-promoting bacteria, Principal Component Analysis (PCA), proline, water deficit

## Abstract

This study aimed to evaluate the synergistic effects of zinc sulfate and *Pseudomonas* spp. in terms of mitigating drought stress in maize (*Zea mays* L.) by analyzing physiological, biochemical, and morphological responses under field conditions. A two-year (2018–2019) field experiment investigated two irrigation levels (optimal and moderate stress) and twelve treatment combinations of zinc sulfate application methods (without fertilizer, soil, foliar, and seed priming) with zinc-solubilizing bacteria (no bacteria, *Pseudomonas fluorescens*, and *Pseudomonas aeruginosa*). Drought stress significantly reduced chlorophyll content, increased oxidative damage, and impaired membrane stability, leading to a 42.4% increase in electrolyte leakage and a 10.9% reduction in leaf area index. However, the combined application of zinc sulfate and *P. fluorescens*, and *P. aeruginosa* mitigated these effects, with seed priming showing the most significant improvements. Specifically, seed priming with zinc sulfate and *P. fluorescens* increased catalase activity by 76% under non-stress conditions and 24% under drought stress. Principal component analysis revealed that treatments combining zinc sulfate and *P. fluorescens*, and *P. aeruginosa* were strongly associated with improved chlorophyll content, carotenoid content, and grain yield while also enhancing osmotic adjustment and antioxidant enzyme activity. These findings highlight the potential of the use of zinc sulfate and *P. fluorescens* as well as *P. aeruginosa* as sustainable strategies for enhancing maize drought tolerance, mainly through seed priming and soil application methods.

## 1. Introduction

Drought stress is one of the most significant abiotic stressors limiting agricultural productivity worldwide, particularly in regions dependent on rain-fed agriculture. Maize (*Zea mays* L.), a staple crop for over 1.2 billion people globally, is highly susceptible to water scarcity, adversely affecting its growth, yield, and nutritional quality [1]. With climate change exacerbating the frequency and severity of droughts, developing strategies to enhance maize resilience to water stress has become a critical research priority. Recent studies highlight the potential of integrating micronutrient management and microbial interventions to mitigate drought-induced losses in crops [2,3]. Among these, zinc (Zn) and plant-growth-promoting rhizobacteria (PGPR) supplementation, particularly with *Pseudomonas* spp., have emerged as promising approaches to improve drought tolerance in maize.

Zinc is an essential micronutrient in numerous physiological processes, including enzyme activation, chlorophyll synthesis, and hormone regulation [4]. However, its availability in soils is often limited, especially in calcareous soils with high pH, which are prevalent in drought-prone regions [5]. Zinc deficiency exacerbates the adverse effects of drought stress by impairing root development, reducing photosynthetic efficiency, and increasing oxidative damage [6]. Recent research has demonstrated that Zn application, particularly zinc sulfate, can enhance drought tolerance by improving water use efficiency, antioxidant enzyme activity, and osmotic adjustment in maize [7,8].

In parallel, PGPR, such as *Pseudomonas fluorescens* and *Pseudomonas aeruginosa*, have gained attention for enhancing plant growth and stress tolerance through multiple mechanisms. These include the production of phytohormones, siderophores, and exopolysaccharides, as well as the induction of systemic resistance [9,10]. *Pseudomonas* spp., particularly *P. fluorescens*, are highly effective due to their metabolic versatility and ability to enhance soil health [11]. These microorganisms strengthen essential nutrient availability and improve nutrient use efficiency [12]. Recent studies have shown that co-inoculation of maize with *Pseudomonas* spp. and Zn application can synergistically improve drought tolerance by enhancing root architecture, nutrient uptake, and antioxidant defense systems [13,14].

Despite these advancements, significant research gaps remain. First, most studies have focused on the individual effects of Zn [4,6] or *Pseudomonas* spp. [9,10], with limited exploration of their combined impact on maize under drought stress. Second, the physiological mechanisms underlying the synergistic effects of Zn and *Pseudomonas* spp.—particularly their roles in ROS scavenging pathways [15] and nutrient uptake regulation [14]—require further elucidation. Third, there is a lack of field-based studies validating the efficacy of these interventions under real-world drought conditions. Addressing these gaps is crucial for developing scalable and sustainable strategies to enhance maize productivity in drought-affected regions.

In a previous study published in *Agricultural Water Management*, we investigated the effects of zinc sulfate and *Pseudomonas* spp. on maize yield and yield components under drought stress [16]. While that study demonstrated significant improvements in grain yield and water use efficiency, the underlying physiological and biochemical mechanisms driving these improvements remained unexplored. To address this gap, the current study focuses on maize’s physiological, biochemical, and morphological responses to zinc sulfate and *Pseudomonas* spp. under drought stress, including chlorophyll content, antioxidant enzyme activity, osmotic adjustment, and membrane stability. Additionally, Principal Component Analysis (PCA) was employed to identify the key variables driving the treatments’ differences and visualize the relationships between physiological and biochemical traits.

## 2. Materials and Methods

### 2.1. Site Description and Experimental Design

**Figure 1:**
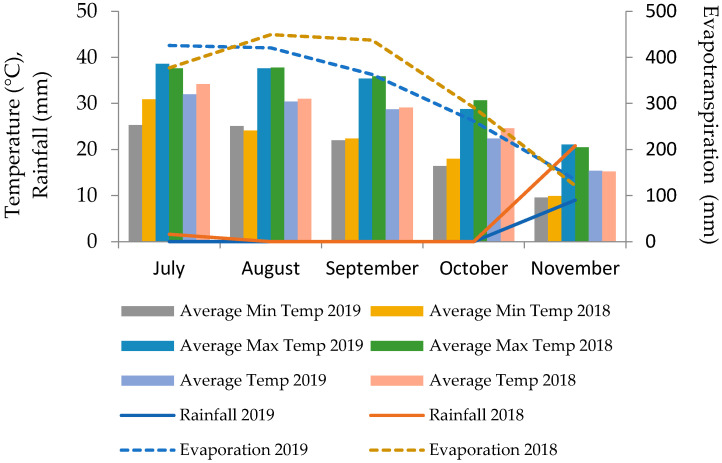
Simplified climatic conditions during the maize growing season (July–November 2018–2019). Monthly averages for temperature (°C), precipitation (mm), and reference evapotranspiration (ET_0_, mm) align with data from [16].

The field experiment was conducted during the 2018 and 2019 growing seasons in Neyriz, Fars, Iran, as described in our previous study [16]. Experimental design (e.g., irrigation levels, treatment combinations) followed the same protocols to ensure comparability. Climatic data (Figure 1) cover the maize growing season (July–November 2018–2019), with planting occurring in early August and harvest conducted by late November, as detailed in [16]. The experimental design was a split-plot arrangement within a randomized complete block design (RCBD) with three replications. The main plots were assigned to two irrigation levels: optimal irrigation and moderate drought stress (60% crop water requirement; see Section 2.2 for details). The subplots consisted of four zinc sulfate (ZnSO_4_·7H_2_O, 23% Zn; Sigma-Aldrich, St. Louis, MO, USA) application methods (without fertilizer, foliar application, soil application, and seed priming) combined with three levels of Zn-solubilizing bacteria (no bacteria, *Pseudomonas fluorescens*, and *Pseudomonas aeruginosa*), resulting in 12 treatment combinations. The control treatment comprised plants receiving neither Zn supplementation nor bacterial inoculation.

### 2.2. Field Preparation, Planting, and Management

Field preparation was performed by plowing, double disking, and furrow formation using a furrower, respectively. Plots were established with five 4 m long rows spaced 50 cm apart. The distance between blocks was 2 m, while the distance between main plots (irrigation treatments) and subplots (fertilizer treatments) was 1.5 m and 0.5 m, respectively. The hybrid maize variety SC 538 (mid-season) was used, with seeds obtained from the Fars Maize Cultivation Development Company, Shiraz, Iran. Nitrogen fertilizer was applied as urea at a 400 kg·ha^−1^ rate. In contrast, based on soil test results, phosphorus and potassium fertilizers were applied as triple superphosphate and potassium sulfate. One-third of the nitrogen fertilizer and all phosphorus and potassium fertilizers were applied at planting, with the remaining nitrogen applied in two split doses (at the 6–7-leaf stage and two weeks before tasseling). Seeds were planted manually in early August at a 4–6 cm depth, with 2–3 seeds per hill spaced 30 cm apart on 50 cm ridges [17]. Uniform irrigation was applied to all plots until plant establishment (3–4 leaf stage), after which drought stress treatments were imposed. The drought stress treatments included no stress (100% crop water requirement based on evapotranspiration) and moderate stress (60% crop water requirement) [18], and were applied after 60 mm of cumulative evaporation from a Class A evaporation pan. The irrigation water requirement was calculated using Formulas (1)–(3) [19]:

I_n_ = 0.623 × A × K_c_ × ET_o_ × IE^−1^ (1)

*In* is the irrigation water volume (m^3^), 0.623 is a constant coefficient, *A* is the plot area (m^2^), *Kc* is the crop coefficient, *ET*_0_ is the reference evapotranspiration (mm), and *IE* is the irrigation efficiency (90% for drip irrigation) [20]. Reference evapotranspiration (*ET*_0_) was calculated using a Class A evaporation pan [21] and the pan coefficient (*Kp*) was determined as follows [22]:

ET_o_ = E_pan_ × K_p_ (2)

*Epan* is the evaporation from the Class A pan (mm·day^−1^), and *Kp* is the pan coefficient (dimensionless). The pan coefficient was calculated as follows:

Kp = 0.482 + 0.024 ln(F) − 0.000376 U + 0.0045 RH(3)

*U* is the average daily wind speed at 2 m height (km/day^−1^), *RH* is the average daily relative humidity (%), and *F* is the distance of the pan from vegetation (m). The crop coefficients for maize ranged from 0.36 to 0.58 during early growth, from 0.71 to 1.13 during mid-growth, and from 0.98 to 0.65 at harvest [23]. Soil moisture was measured before and after irrigation.

### 2.3. Physiological, Biochemical, and Morphological Measurements

Measurements were conducted at specific growth stages during both growing seasons (2018: 5 August–15 November; 2019: 8 August–18 November) as follows:

Biochemical traits: Chlorophyll and carotenoid content were measured spectrophotometrically following the extraction of fresh leaf tissue in 80% acetone (*v*/*v*) (Merck, Darmstadt, Germany), with absorbance measured at 663, 645, and 470 nm (UV-1800 spectrophotometer, Shimadzu, Kyoto, Japan) using Arnon’s method [24]. Proline content was quantified via ninhydrin reagent (Sigma-Aldrich, St. Louis, MO, USA) at 520 nm [25]. Soluble sugars were extracted in 80% ethanol (Merck, Darmstadt, Germany) at 80 °C for 1 h (water bath: WB-200, Jeio Tech, Seoul, Republic of Korea); then, the supernatant was reacted with anthrone reagent (Sigma-Aldrich, St. Louis, MO, USA) and measured at 620 nm [26]. Malondialdehyde (MDA) content was estimated through the thiobarbituric acid (TBA; Sigma-Aldrich, St. Louis, MO, USA) reaction at 532/600 nm [27].

Antioxidant enzyme activities were determined as follows: catalase (CAT)—H_2_O_2_ decomposition at 240 nm [28]; superoxide dismutase (SOD)—NBT reduction at 560 nm [29]; peroxidase (POD)—guaiacol oxidation at 470 nm [30].

Physiological traits: Membrane stability or electrolyte leakage (EL) was measured using the method of [31]. Glycine betaine content was analyzed using the potassium iodide spectrophotometric method [32].

Morphological traits: Leaf area index (LAI) was measured weekly based on tasseling (45 DAP) through pollination. All biochemical, physiological, and morphological measurements were performed at the pollination stage (62–65 DAP).

### 2.4. Statistical Analysis

Data normality and homogeneity of variances (Bartlett’s test) were assessed before analysis. If Bartlett’s test yielded non-significant results, a two-year combined analysis (three-way ANOVA) was performed for the two-year data using PROC GLM in SAS software version 9.1 (SAS Institute, Cary, NC, USA). Where higher-order interactions (e.g., Year × Drought × Treatment) were significant (*p* < 0.05), lower-order effects (main or two-way interactions) were not interpreted separately, as they were biologically nested within the three-way interaction. Mean comparisons were only conducted using the LSD test (*p* < 0.05) for significant interactions, and graphs were prepared using Microsoft Excel (version 2013). Principal Component Analysis (PCA) was employed to identify the key variables driving the differences among treatments and to visualize the relationships between physiological and biochemical traits. The PCA was performed using the factoextra and ggplot2 packages in the R software (version 4.1.2) to identify the key variables driving the treatments’ differences and visualize the relationships among physiological, morphological, and biochemical traits.

## 3. Results

### 3.1. Chlorophyll and Carotenoid Content

The results showed significant effects of year × drought stress × zinc sulfate–bacteria combination on chlorophyll *a* content (*p* ≤ 0.01) (Table 1). Chlorophyll *a* peaked in 2019 for *P. fluorescens*-treated plants (1.93 mg·g^−1^ FW), though the results were statistically similar to those obtained for control (1.61 mg·g^−1^ FW), Zn priming (1.58 mg·g^−1^ FW), and soil Zn (1.71 mg·g^−1^ FW) treatments under optimal conditions (Table 2), indicating comparable efficacy between microbial and Zn-only approaches in the absence of stress, while the lowest values were recorded in the treatment without zinc sulfate and bacteria under drought stress in 2018. In the control treatment, drought stress reduced chlorophyll *a* content by 13% and 4% in 2018 and 2019, respectively. While the *P. fluorescens* treatment under non-stressed conditions in 2019 showed numerically higher chlorophyll *a* content (1.93 mg·g^−1^ FW) compared to the control (1.61 mg·g^−1^ FW), this 20% increase was not statistically significant (*p* > 0.05, LSD test; Table 2). However, the consistent directional trend across replicates suggests that there was a biologically meaningful, albeit non-significant, enhancement effect.

Chlorophyll *b* content was significantly affected by year (*p* ≤ 0.01) and drought stress (*p* ≤ 0.05) (Table 1). Plants in 2019 produced 22% more chlorophyll *b* than those in 2018 (Table 3). Drought stress increased chlorophyll *b* content by 9.5% compared to non-stress conditions (Table 4).

Chlorophyll *ab* and carotenoid content were significantly influenced by the three-way interaction of year × drought stress × zinc sulfate–bacteria combination (*p* ≤ 0.01) (Table 1). The numerically highest total chlorophyll content (2.50 mg·g^−1^ FW) was observed in the *Pseudomonas fluorescens* treatment under non-stressed conditions in 2019, which was 19% higher than the control. However, this difference was not statistically significant. The lowest total chlorophyll content (1.03 mg·g^−1^ FW) was recorded in the treatment without zinc sulfate and bacteria under drought stress in 2018 (Table 2). While numerically lower than most treatments, this value did not differ significantly (*p* > 0.05) from soil Zn application under drought (1.13 mg·g^−1^ FW), *P. aeruginosa* inoculation under drought (1.33 mg·g^−1^ FW), and control treatments in 2018 (1.13 mg·g^−1^ FW).

The highest carotenoid content was observed in the treatment with *P. aeruginosa* under non-stressed conditions in 2019; it was more than double that of the control. The lowest carotenoid content was recorded in the treatment with soil application of zinc sulfate and *P. aeruginosa* under drought stress in 2018 (Table 2).

### 3.2. Osmoprotectants and Oxidative Stress

Proline content was significantly influenced by the interactions of year × drought stress × zinc sulfate–bacteria combination (*p* ≤ 0.01) (Table 1). The highest proline content (31.40 μmol·g^−1^) was observed in the foliar application treatment with zinc sulfate and no bacteria under drought stress in 2018. However, applying *P. bacteria* reduced proline content, with the soil application of zinc sulfate and *P. fluorescens* under drought stress in 2018 reducing proline content by 54% compared to the control (Table 2).

**Figure 2:**
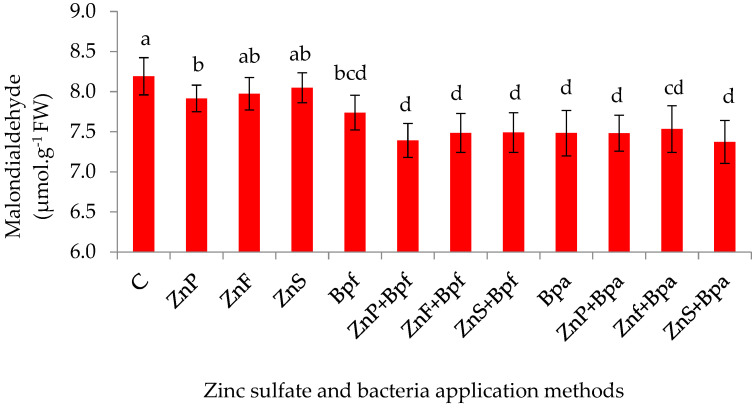
Effect of application methods of zinc sulfate and bacteria on MDA of maize. C (control), ZnP (zinc priming), ZnF (foliar zinc), ZnS (soil zinc), Bpf (*P. fluorescens*), Bpa (*P. aeruginosa*). Values of Bar graphs are expressed as mean ± SE (n = 12). Different letters indicate significant differences (*p* < 0.05, LSD test).

Statistical analysis revealed the significant main effects of both drought stress (*p* ≤ 0.01) and zinc sulfate–bacteria treatments (*p* ≤ 0.01) on malondialdehyde (MDA) content. In contrast, soluble sugar content was predominantly influenced by their interaction (drought × treatment, *p* ≤ 0.01) (Table 1). Drought stress increased MDA content by 19% compared to non-stressed conditions (Table 4). Across both irrigation regimes (main effects), applying zinc sulfate with *Pseudomonas* bacteria significantly reduced MDA content in most treatments (Figure 2). The application of *Pseudomonas* spp. significantly reduced MDA content compared to both the control (8.19 µmol·g^−1^ FW) and all Zn-only treatments (Zn priming: 7.92 ± 0.12; Zn foliar: 7.85 ± 0.15; soil Zn: 7.81 ± 0.18 µmol·g^−1^ FW; *p* < 0.05). The lowest MDA levels were achieved when combining soil Zn and *P. aeruginosa* (7.37 ± 0.09 µmol·g^−1^), demonstrating superior oxidative stress mitigation (Figure 2). The highest soluble sugar content (81.5 mg·g^−1^ FW) was observed in the seed priming treatment with zinc sulfate and *P. fluorescens* under drought stress, with a value 23% higher than the control. The lowest soluble sugar content was recorded in the seed priming treatment with zinc sulfate and *P. aeruginosa* under non-stressed conditions (Table 5).

Glycine betaine content (Table 1) and membrane electrolyte leakage (Table 6) were only significantly influenced by drought stress (*p* ≤ 0.01). Drought stress significantly increased glycine betaine, by 24%, and membrane electrolyte leakage, by 42% (indicating reduced membrane stability), compared to non-stressed conditions (Table 4).

### 3.3. Antioxidant Enzyme Activity

Peroxidase activity was significantly affected by the three-way interaction of year × drought stress × zinc sulfate–bacteria combination (*p* ≤ 0.05) (Table 6). The highest peroxidase activity was observed in treatments under drought stress and the control without zinc sulfate and bacteria in 2019, while the lowest activity was recorded in the *P. fluorescens* without zinc sulfate under non-stressed conditions in the same year (Table 2).

Catalase activity was significantly influenced by year (*p* ≤ 0.01) and the interaction of drought stress × zinc sulfate–bacteria combination (*p* ≤ 0.05) (Table 6). Mean comparison data revealed a 39% reduction in catalase activity in 2019 compared to 2018, with values of 183.10 and 112.33 mM·g^−1^ Fw/min, respectively (Table 3). The highest catalase activity (202.33 mM·g^−1^ Fw/min) was observed in the seed priming treatment with zinc sulfate and *P. fluorescens* under drought stress. The lowest (106.33 mM·g^−1^ Fw/min) was recorded in the control treatment without zinc sulfate and bacteria under non-stressed conditions (Table 5).

**Figure 3:**
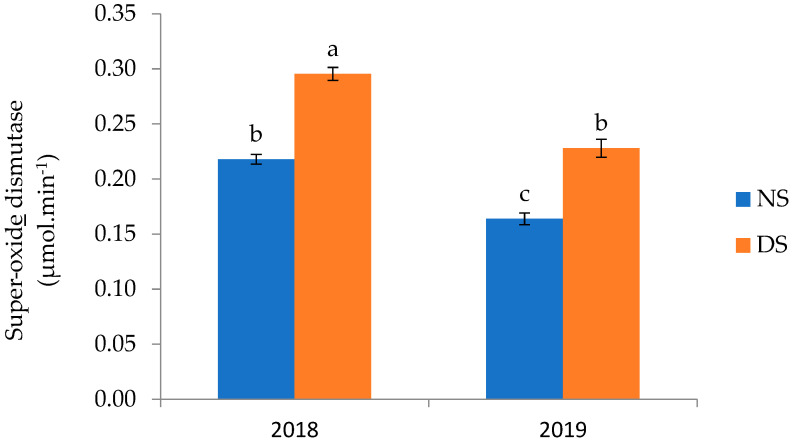
The mean comparison of the impact of the interaction between year and drought stress on the superoxide dismutase activity of maize. NS (no stress, 100% irrigation), DS (drought stress, 60% irrigation). Values of bar graphs are expressed as mean ± SE (n = 36). Different letters indicate significant differences (*p* < 0.05, LSD test). Data derived from significant two-way interactions (year × drought) following ANOVA in Table 6.

Superoxide dismutase (SOD) activity was significantly affected by the interaction of year × drought stress (*p* ≤ 0.01) (Table 6). The highest SOD activity (0.295 unite·min^−1^) was observed under drought stress in 2018, which was 35% higher than the non-stressed treatment. The lowest SOD activity was recorded in the non-stressed treatment in 2019 (Figure 3).

### 3.4. Leaf Area Index (LAI) and Grain Yield

**Figure 4:**
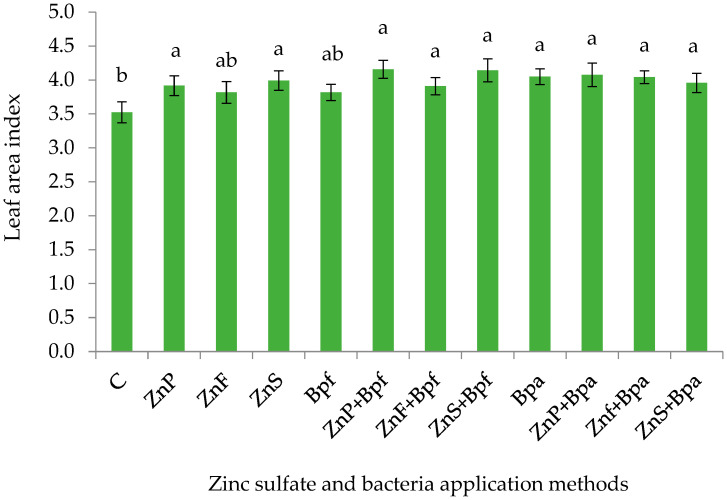
The effect of zinc sulfate application methods and bacteria on maize LAI. C (control), ZnP (zinc priming), ZnF (foliar zinc), ZnS (soil zinc), Bpf (*P. fluorescens*), and Bpa (*P. aeruginosa*). Values of Bar graphs are expressed as mean ± SE (n = 12). Different letters indicate significant differences (*p* < 0.05, LSD test).

LAI was significantly influenced by year, drought stress, and the combined application of zinc sulfate and bacteria (*p* ≤ 0.05) (Table 6). Mean comparison data revealed a significant increase in LAI in the second year and an 11% reduction under drought stress compared to non-stressed conditions (Table 3 and Table 4). Numerically, the highest LAI value (4.16) was recorded in the seed priming treatment with zinc sulfate and *P. fluorescens*, though this did not differ significantly from most other treatments. At the same time, the lowest (3.52) was recorded in the control treatment without zinc sulfate and bacteria (Figure 4). The observed range across all treatments (3.52–4.16) suggests that there were limited treatment effects on LAI under the experimental conditions (Figure 4).

Grain yield responses to treatments were reported in our prior study [16], where synergistic effects of zinc and *Pseudomonas* spp. significantly improved yield under drought (e.g., +36.9% with *P. aeruginosa*). Here, we focus on linking these yield outcomes to physiological mechanisms (e.g., antioxidant activity, osmotic adjustment).

The lack of Zn and microbial interventions in these treatments resulted in lower chlorophyll content, reduced antioxidant enzyme activity, and ultimately lower grain yield.

### 3.5. Principal Component Analysis (PCA)

**Figure 5:**
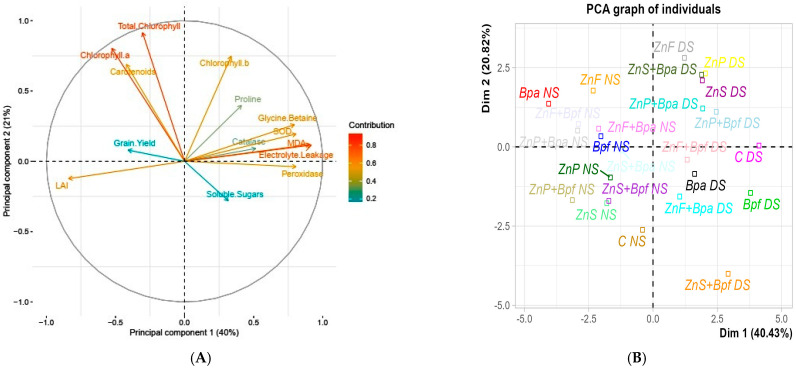
Principal Component Analysis (PCA) biplot illustrating the relationships between physiological and biochemical traits under different treatments of zinc sulfate and *Pseudomonas* spp. in maize under drought stress: (**A**) loadings of physiological and biochemical traits; (**B**) treatment scores (codes as in Table 2). Arrows indicate trait contributions to PC1/PC2.

**Figure 6:**
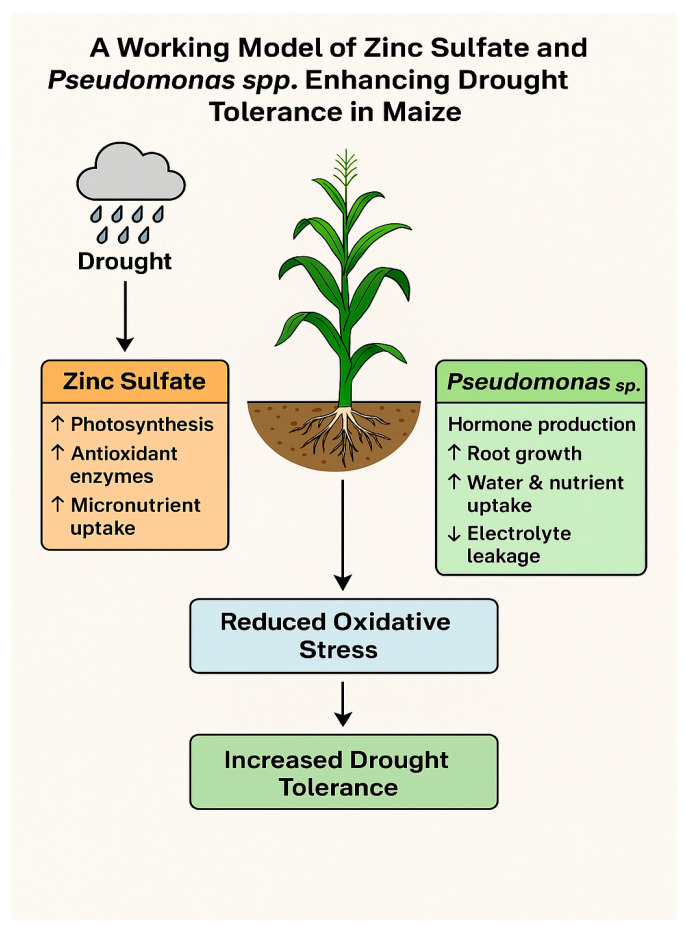
Working model illustrating synergistic effects of zinc sulfate and *Pseudomonas* on maize drought tolerance.

The first two principal components (PC1 and PC2) explained 61% of the total variance in the dataset, with PC1 accounting for 40% and PC2 for 21% (Figure 5). The biplot (Figure 6) illustrates the relationships between the treatments and the measured traits, providing insights into the underlying mechanisms of drought stress mitigation by zinc sulfate and *Pseudomonas* spp.

Principal Component Analysis (PCA) revealed that treatments combining zinc sulfate and *Pseudomonas* spp. clustered on the positive side of PC1, primarily due to their strong association with enhanced antioxidant enzyme activities (catalase, peroxidase) and osmotic adjustment (proline, soluble sugars) (Figure 6). While grain yield and photosynthetic pigments (chlorophyll ab, carotenoids) contributed to PC2 (12% of variance), their loadings were less dominant in the PC1 dimension.

On the other hand, control treatments (without zinc sulfate and *Pseudomonas* spp.) and drought-stressed treatments clustered on the negative side of PC1, showing strong positive loadings (>0.7) with oxidative stress markers (MDA: 0.82; electrolyte leakage: 0.79) (Figure 5). This axis explained 37% of the total variance, effectively separating stress-responsive from stress-mitigating treatments. These treatments were also associated with increased proline and soluble sugar content, reflecting the plant’s osmotic adjustment response to drought stress.

## 4. Discussion

Drought stress led to marked reductions in maize growth and yield, primarily due to diminished chlorophyll content, elevated oxidative stress, and compromised membrane stability. These detrimental effects are consistent with previous reports linking water deficits to impaired photosynthesis and increased lipid peroxidation. However, the application of zinc sulfate and *Pseudomonas* spp. appeared to counteract these stress responses by enhancing antioxidant defense mechanisms, maintaining chlorophyll stability, and supporting osmotic adjustment. These findings suggest that integrating micronutrient supplementation with PGPR inoculation could be a promising strategy for bolstering maize resilience under drought conditions. While this study focused on moderate drought stress (60% ETc) to reflect common field conditions, future research should incorporate severe stress treatments (<50% ETc) to evaluate the upper limits of the observed protective effects.

### 4.1. Chlorophyll and Carotenoid Content

Unlike chlorophyll a, chlorophyll b content increased slightly under drought stress (from 0.42 to 0.46 mg g^−1^ FW, Table 4), which may reflect an adaptive adjustment to maintain light harvesting under suboptimal conditions. This observation partially contrasts with previous reports attributing drought-induced declines in chlorophyll to enhanced reactive oxygen species (ROS) accumulation and pigment degradation [33,34]. However, the increase in chlorophyll b may indicate a compensatory mechanism by which plants attempt to preserve photosynthetic function under stress.

For instance, Abdel-Motagally and El-Zohri [33] demonstrated a 35% decrease in chlorophyll content in wheat (*Triticum aestivum* L.) under similar drought conditions, linking it to ROS-induced thylakoid membrane damage [33].

Under drought stress conditions, seed priming and soil application of zinc sulfate in combination with *P. fluorescens* significantly improved chlorophyll *a* and total chlorophyll content (Table 2). This suggests that these treatments have a protective role in preserving photosynthetic pigments under water-deficit stress. Zinc stabilizes chloroplast membranes and promotes chlorophyll biosynthesis [5], while *P. fluorescens* enhances micronutrient solubilization and stimulates root growth, improving nutrient and water uptake under stress [9,13]. The observed improvements in chlorophyll content under drought conditions indicate that the synergistic application of zinc sulfate and *Pseudomonas* spp. may delay chlorophyll degradation and sustain photosynthetic efficiency in maize. Also, applying zinc sulfate and *P. fluorescens* significantly improved chlorophyll content, particularly with seed priming under non-stressed conditions. This corroborates findings by Ghasemi et al. [5], who reported that Zn stabilizes chloroplast membranes by upregulating *LHCB* (light-harvesting complex) genes, while *Pseudomonas* spp. enhance magnesium uptake—a central component of chlorophyll [5,9].

Carotenoids play a critical role in photoprotection and scavenging reactive oxygen species (ROS), particularly under drought-induced oxidative stress. In the present study, although carotenoid content did not consistently increase across all treatments under drought stress, applications involving zinc sulfate combined with *P. fluorescens* or *P. aeruginosa* generally helped maintain carotenoid levels at values comparable to or higher than the control (Table 2). This suggests that there was a stabilizing effect on pigment retention under stress. Previous studies have also reported that Zn enhances carotenoid synthesis by supporting antioxidant metabolism [35]. At the same time, *Pseudomonas* spp. may protect pigment integrity through improved water and nutrient uptake and reduced oxidative load [13,36].

However, seed priming with zinc sulfate and *P. aeruginosa* under non-stressed conditions resulted in the highest carotenoid content. This parallels the work of Silva et al. [35], who observed a 1.8-fold increase in carotenoids in sugarcane (*Saccharum officinarum* L.) treated with Zn-solubilizing bacteria, which was attributed to enhanced precursor (phytoene) synthesis [34]. A recent meta-analysis by Gu et al. [3] further suggests that microbial consortia (including *Pseudomonas*) upregulate carotenoid biosynthesis genes (*PSY*, *LCYB*) under abiotic stress.

The observed synergistic effects of zinc sulfate and *Pseudomonas* spp. likely operate through multiple interconnected pathways. First, Zn acts as an essential cofactor for copper/Zn superoxide dismutase (Cu/Zn-SOD), a critical enzyme that scavenges reactive oxygen species (ROS) and protects chlorophyll molecules from oxidative degradation [6]. Concurrently, Pseudomonas-derived siderophores enhance iron bioavailability in the rhizosphere [9], facilitating iron incorporation into chlorophyll biosynthesis pathways and maintaining photosynthetic efficiency under stress conditions. Furthermore, microbial exopolysaccharides produced by *Pseudomonas* strains contribute to soil moisture retention and osmotic adjustment, helping maintain leaf turgor pressure and delaying drought-induced stomatal closure [10]. These combined mechanisms—from molecular-level antioxidant protection to whole-plant water regulation—collectively enhance maize resilience to water deficits while improving nutrient use efficiency.

### 4.2. Osmoprotectants and Oxidative Stress

The accumulation of proline and soluble sugars under drought stress is an osmotic adjustment mechanism to maintain cell turgor and protect cellular structures [37]. In this study, proline content, as a non-enzymatic antioxidant, increased significantly under drought stress, particularly in treatments without bacterial inoculation. However, applying *Pseudomonas* bacteria reduced proline levels, suggesting that there was improved stress tolerance and membrane stability. This aligns with the work of Shoresh et al. [38], who found that *Pseudomonas* spp. enhance water use efficiency and reduce the need for osmotic adjustment under stress conditions.

Soluble sugar content exhibited variation under drought stress, with significant increases observed in treatments involving seed priming with zinc sulfate and *P. fluorescens* (Table 5). This suggests that these treatments enhanced osmotic adjustment by promoting carbohydrate accumulation, critical for maintaining cell turgor and mitigating stress-induced damage. In contrast, other treatments did not show consistent increases in soluble sugars, indicating that the effect of Zn and *Pseudomonas* on sugar metabolism depends on the specific application method and interactions with the plant’s stress response mechanisms.

This is consistent with Hinojosa et al. [36], who reported that *Pseudomonas* strains enhance carbohydrate metabolism and sugar accumulation, thereby improving stress tolerance. Zinc, as a cofactor for enzymes involved in carbohydrate metabolism, further supports this process [39]. These findings suggest combining zinc sulfate and *P. fluorescens* can enhance maize’s ability to adapt to drought stress through improved carbohydrate accumulation.

Malondialdehyde (MDA) content, a marker of lipid peroxidation, increased under drought stress, indicating oxidative damage to cell membranes. However, applying zinc sulfate and *Pseudomonas* bacteria significantly reduced MDA levels, likely by enhancing antioxidant enzyme activity and reducing ROS production. Similar results were reported by Azeem et al. [40], who demonstrated that Zn and microbial inoculants improve membrane stability under drought stress.

Glycine betaine, a well-known osmoprotectant, is crucial in osmotic adjustment and cellular protection under drought stress conditions [32,41]. In this study, glycine betaine content significantly increased under drought stress, aligning with previous findings highlighting its role in stabilizing cellular structures and protecting enzymes and proteins from dehydration-induced damage [41,42]. Glycine betaine accumulates in the cytoplasm and chloroplasts, where it helps maintain membrane integrity and protein functionality, thereby enhancing the plant’s ability to cope with osmotic stress [32]. This increase in glycine betaine content reflects a key adaptive response to drought, as it mitigates the detrimental effects of water deficit by preserving cellular homeostasis [43]. This finding aligns with Ali et al. [41], who reported that glycine betaine protects chloroplasts under stress, supporting our result.

Electrolyte leakage, a marker of membrane damage, was significantly increased by drought stress, indicating oxidative damage to cell membranes due to the accumulation of reactive oxygen species (ROS) and lipid peroxidation [27]. However, the application of zinc sulfate and *Pseudomonas* spp. did not significantly affect EL under drought stress (Table 6), suggesting that these treatments did not reduce membrane damage through the direct stabilization of membrane integrity. This is consistent with previous studies’ findings, where drought stress’s primary effect on electrolyte leakage was attributed to oxidative damage, and treatments like Zn and PGPR were found to mitigate oxidative stress through other mechanisms, such as by enhancing antioxidant enzyme activity [3,7].

### 4.3. Antioxidant Enzyme Activity

The differential enzyme responses suggest the temporal modulation of antioxidant defenses [15]. While catalase and peroxidase provided consistent drought protection, superoxide dismutase (SOD) effectiveness depended on yearly conditions, possibly due to fluctuations in environmental factors such as temperature or evapotranspiration. This aligns with the work of Ghanbari et al. [44] findings of enzyme-specific stress adaptation thresholds, where the activity of antioxidant enzymes like SOD varied with environmental stress intensity, highlighting that different enzymes may operate under distinct stress conditions and adapt differently over time.

The highest catalase activity was observed in the seed priming treatment with zinc sulfate and *P. fluorescens*. This aligns with the work of Ghanbari et al. [44], who reported that microbial inoculants enhance antioxidant enzyme activity by inducing plant systemic resistance. Similarly, Vazin [8] found that Zn improves the activity of SOD and catalase by stabilizing enzyme structures and enhancing their efficiency.

### 4.4. Leaf Area Index (LAI) and Grain Yield

The yield improvements reported in [16] align with the observed physiological resilience (e.g., sustained chlorophyll content, reduced MDA) under combined zinc–*Pseudomonas* treatments (Section 3.1, Section 3.2, Section 3.3), suggesting that osmotic and antioxidant responses underlie yield stability. However, while drought stress significantly reduced the leaf area index (LAI), no significant interaction between drought stress and the treatments was observed for LAI. This suggests that the effects of these treatments on leaf expansion and photosynthetic capacity may not be as pronounced as their impact on grain yield under drought stress. The observed improvement in grain yield aligns with previous studies where nutrient management and microbial inoculation enhanced crop productivity under stress, possibly through improved nutrient uptake and resilience mechanisms [3,7]. This is consistent with Ghanbari et al. [44], who reported that Zn and microbial inoculants enhance nutrient uptake, root development, and photosynthetic efficiency, leading to higher yields.

### 4.5. Principal Component Analysis (PCA)

Principal Component Analysis (PCA) revealed the distinct clustering of treatments, with those combining zinc sulfate and *Pseudomonas* spp.—particularly seed priming and soil application methods—strongly associated with improved chlorophyll content, carotenoid, and grain yield. These findings suggest that the synergistic combination of Zn and PGPR significantly enhances maize performance under drought stress. Previous studies support this, showing that Zn and PGPR can enhance photosynthetic efficiency and nutrient uptake, thus improving crop resilience to stress conditions [9]. The positive correlation between these treatments and PC1 underscores their pivotal role in mitigating drought stress by enhancing key physiological processes, such as photosynthesis and nutrient assimilation.

In contrast, the control treatments (without zinc sulfate and *Pseudomonas* spp.) and drought-stressed treatments were clustered on the negative side of PC1, indicating the existence of a strong association with oxidative stress markers, including malondialdehyde (MDA) and electrolyte leakage. These treatments also exhibited increased proline and soluble sugar content, reflecting the plant’s osmotic adjustment mechanisms in response to water deficits [37]. However, without Zn and microbial intervention, these treatments showed reduced chlorophyll content, lower antioxidant enzyme activity, and diminished grain yield, highlighting the importance of integrated nutrient and microbial management in environments prone to drought stress [2].

The separation of treatments along PC2 further emphasized the contribution of *Pseudomonas* spp. to drought stress mitigation. Treatments inoculated with P. fluorescens and *P. aeruginosa* exhibited higher scores on PC2, which were strongly associated with enhanced antioxidant enzyme activities (such as catalase, peroxidase, and superoxide dismutase), as well as improved osmotic adjustment mechanisms like increased proline and soluble sugar accumulation. These results suggest that *Pseudomonas* spp. not only improve nutrient availability but also actively stimulate plant defense responses by enhancing enzymatic antioxidant systems. Consequently, plants treated with *Pseudomonas* spp. experienced reduced oxidative damage under drought stress, reinforcing the key role of microbial inoculants in improving stress resilience.

This aligns with the findings of Shoresh and Harman [38], who reported that PGPR enhances plant stress tolerance by inducing systemic resistance and activating antioxidant defense mechanisms. Recent studies further support the synergistic effect of zinc sulfate and *Pseudomonas* spp. in reducing oxidative damage and maintaining cellular integrity under drought stress [8].

The insights gained from the PCA provide a clearer understanding of the physiological and biochemical mechanisms driving the synergistic effects of zinc sulfate and *Pseudomonas* spp. in mitigating drought stress in maize. The strong association of these treatments with improved chlorophyll content, carotenoid, and grain yield highlights their potential as sustainable strategies for improving maize productivity in drought-affected regions. Moreover, the close relationship between these treatments and enhanced antioxidant enzyme activity emphasizes their role in reducing oxidative stress and maintaining cellular homeostasis under water-deficient conditions.

Despite this study’s strengths, such as the comprehensive evaluation of multiple physiological and biochemical traits under field conditions, there are limitations to consider. The experiment was conducted in a specific geographic region with calcareous soils, which may limit the generalizability of the results to other soil types and climates. Furthermore, the study focused on two *Pseudomonas* species, and further research is needed to explore the effects of other microbial inoculants on maize drought tolerance.

### 4.6. Proposed Mechanistic Model for Drought Tolerance Enhancement

Based on the observed physiological and biochemical responses, we propose a working model (Figure 6) that illustrates the synergistic role of zinc sulfate and *Pseudomonas* spp. in enhancing drought tolerance in maize. Zinc sulfate contributes to drought mitigation by improving chlorophyll biosynthesis, stabilizing chloroplast membranes, enhancing antioxidant enzyme activity, and promoting osmotic adjustment. Concurrently, *P. fluorescens* and *P. aeruginosa* act as plant-growth-promoting rhizobacteria (PGPR) that enhance root development, nutrient solubilization (including Zn), and the production of phytohormones and exopolysaccharides, effects which collectively improve water uptake and stress resilience. The co-application of Zn and *Pseudomonas* spp. amplifies these effects by increasing chlorophyll and carotenoid content to sustain photosynthesis under drought conditions; enhancing the activity of catalase, peroxidase, and superoxide dismutase to detoxify reactive oxygen species (ROS); reducing membrane lipid peroxidation and electrolyte leakage; and improving proline and soluble sugar accumulation for osmoprotection. These effects ultimately lead to improved grain yield and water use efficiency.

This integrative model provides a conceptual framework to understand how Zn nutrition and microbial inoculation can interact to induce systemic drought tolerance mechanisms in maize.

## 5. Conclusions

This study demonstrates that the combined application of zinc sulfate and *Pseudomonas* spp. (particularly strain *fluorescens*) significantly enhances maize drought tolerance through synergistic physiological and biochemical mechanisms. Our findings reveal that seed priming with zinc sulfate and *P. fluorescens* increased catalase activity by 76% under optimal conditions and maintained a 24% improvement under drought stress. These physiological improvements were associated with the enhanced drought tolerance reported in our prior study [16], where combined zinc–*Pseudomonas* treatments demonstrated significant field efficacy. The soil application of zinc with *P. aeruginosa* showed particularly robust effects on stress resilience parameters, which correlated with improved crop performance under water-limited conditions. The observed improvements in chlorophyll content, antioxidant enzyme activity, and osmotic adjustment (proline and soluble sugar accumulation) collectively validate the role of Zn as a redox regulator and *Pseudomonas* as a rhizosphere modulator under water deficit. Notably, the PCA further confirmed that treatments combining Zn and *Pseudomonas* clustered strongly with yield-related traits, suggesting their agricultural scalability. These results provide actionable insights for farmers in arid regions, where targeted microbial inoculants paired with Zn nutrition could serve as a sustainable alternative to conventional drought mitigation strategies. Future research should explore the field-scale validation and economic feasibility of these treatments across diverse agroecological zones.

## Figures and Tables

**Table 1:** Combined analysis of variance (mean squares) for physiological and biochemical traits of maize under drought stress: Malondialdehyde (MDA, μmol·g^−1^ FW), glycine betaine (μg·g^−1^ DW), soluble sugars (mg·g^−1^ FW), proline (μmol·g^−1^), carotenoids (mg·g^−1^ FW), and chlorophyll *a*, *b*, and *ab* (mg·g^−1^ FW).

S.O.V.	D.F.	Mean Squares							
		Chlorophyll *a*	Chlorophyll *b*	Chlorophyll *ab*	Carotenoid	Proline	Soluble Sugars	Glycine Betaine	MDA
Year (Y)	1	3.673 **^1^	0.444 **	6.376 **	1.247 **	686.44 **	283.36 ^ns^	0.040 ^ns^	0.183 ^ns^
Replicate (R) × Y	4	0.06	0.013	0.152	0.061	15.07	105.4	0.283	0.069
Drought (D)	1	0.380 **	0.062 *	0.117 ^ns^	0.054 ^ns^	464.40 **	476.69 *	5.210 **	64.26 **
D × Y	1	0.0003 ^ns^	0.010 ^ns^	0.008 ^ns^	0.250 ^ns^	21.01 ^ns^	156.25 ^ns^	0.012 ^ns^	0.011 ^ns^
R × Y × D	4	0.044	0.006	0.058	0.006	8.27	250.14	0.064	0.274
Zn − Bacteria (ZB)	11	0.136 **	0.019 ^ns^	0.167 *	0.098 **	37.31 **	1244.98 **	0.196 ^ns^	0.859 **
Y × ZB	11	0.293 **	0.014 ^ns^	0.410 **	0.081 **	42.80 **	127.56 ^ns^	0.124 ^ns^	0.087 ^ns^
D × ZB	11	0.094 ^ns^	0.020 ^ns^	0.177 *	0.041 ^ns^	87.47 **	493.74 **	0.230 ^ns^	0.182 ^ns^
Y × D× ZB	11	0.214 **	0.011 ^ns^	0.284 **	0.85 **	54.10 **	80.17 ^ns^	0.200 ^ns^	0.064 ^ns^
Error	88	0.053	0.0107	0.082	0.024	11.7	143.58	0.127	0.201
C.V. (%)		17.38	23.35	16.22	17.5	17.66	21.85	19.94	5.84

^1^ *, ** and ^ns^: significant at 5, 1% of probability and non-significant, respectively.

**Table 2:** The impact of year, drought stress, and the application of zinc sulfate and solvent bacteria on some physiological traits of maize.

Treatment		Chlorophyll *a* (mg·g^−1^ FW)		Chlorophyll *ab* (mg·g^−1^ FW)		Carotenoid (mg·g^−1^ FW)		Proline (μmol·g^−1^)		Peroxidase Activity (unite·min^−1^)	
		2018	2019	2018	2019	2018	2019	2018	2019	2018	2019
NS	C	0.83 ± 0.11 ^cd1^	1.61 ± 0.21 ^ab^	1.13 ± 0.12 ^cd^	2.10 ± 0.31 ^ab^	0.47 ± 0.05 ^c^	0.43 ± 0.06 ^c^	13.3 ± 2.3 ^d^	9.7 ± 0.3 ^e^	0.026 ± 0.003 ^bc^	0.028 ± 0.006 ^bc^
	ZnP	1.19 ± 0.16 ^bc^	1.58 ± 0.12 ^ab^	1.57 ± 0.15 ^c^	1.97 ± 0.15 ^bc^	0.63 ± 0.08 ^bc^	0.37 ± 0.11 ^c^	16.2 ± 1.5 ^cd^	15.3 ± 1.4 ^cd^	0.037 ± 0.003 ^b^	0.023 ± 0.005 ^c^
	ZnF	1.71 ± 0.15 ^ab^	1.40 ± 0.12 ^bc^	2.17 ± 0.13 ^ab^	1.83 ± 0.17 ^bc^	1.00 ± 0.03 ^a^	0.47 ± 0.07 ^c^	14.2 ± 1.4 ^cd^	13.1 ± 1.4 ^d^	0.029 ± 0.003 ^bc^	0.032 ± 0.005 ^bc^
	ZnS	0.86 ± 0.07 ^cd^	1.71 ± 017 ^ab^	1.13 ± 0.11 ^cd^	2.20 ± 0.16 ^ab^	0.47 ± 0.06 ^c^	0.37 ± 0.13 ^c^	30.3 ± 1.5 ^a^	11.7 ± 1.3 ^de^	0.020 ± 0.003 ^c^	0.021 ± 0.002 ^c^
	Bpf	0.92 ± 013 ^cd^	1.93 ± 0.03 ^a^	1.33 ± 0.14 ^cd^	2.50 ± 0.06 ^a^	0.47 ± 0.14 ^c^	0.60 ± 0.04 ^bc^	13.9 ± 1.9 ^d^	15.1 ± 1.1 ^cd^	0.019 ± 0.001 ^c^	0.012 ± 0.001 ^c^
	ZnP+Bpf	1.37 ± 0.29 ^bc^	1.25 ± 0.13 ^bc^	1.67 ± 0.33 ^bc^	1.63 ± 0.08 ^bc^	0.90 ± 0.30 ^ab^	0.37 ± 0.01 ^c^	15.5 ± 1.9 ^cd^	15.4 ± 1.9 ^cd^	0.021 ± 0.003 ^c^	0.022 ± 0.006 ^c^
	ZnF+Bpf	1.46 ± 0.17 ^bc^	1.38 ± 0.12 ^bc^	1.87 ± 0.24 ^bc^	1.83 ± 0.14 ^bc^	0.83 ± 0.13 ^ab^	0.53 ± 0.02 ^bc^	24.1 ± 1.2 ^b^	17.5 ± 1.6 ^cd^	0.023 ± 0.003 ^c^	0.019 ± 0.003 ^bc^
	ZnS+Bpf	1.01 ± 0.15 ^cd^	1.53 ± 0.16 ^b^	1.37 ± 0.21 ^cd^	2.03 ± 0.17 ^b^	0.50 ± 0.10 ^bc^	0.37 ± 0.07 ^c^	21.4 ± 1.5 ^bc^	14.5 ± 2.0 ^cd^	0.039 ± 0.004 ^ab^	0.023 ± 0.005 ^c^
	Bpa	1.52 ± 0.08 ^b^	1.49 ± 0.08 ^bc^	1.93 ± 0.13 ^bc^	1.90 ± 0.07 ^bc^	1.07 ± 0.06 ^a^	0.50 ± 0.02 ^bc^	14.5 ± 1.9 ^cd^	19.6 ± 1.8 ^c^	0.016 ± 0.002 ^c^	0.018 ± 0.004 ^c^
	ZnP+Bpa	1.36 ± 0.11 ^bc^	1.54 ± 0.14 ^b^	1.73 ± 0.11 ^bc^	2.00 ± 0.18 ^bc^	0.90 ± 0.09 ^ab^	1.03 ± 0.07 ^a^	20.7 ± 1.0 ^bc^	20.4 ± 2.6 ^bc^	0.038 ± 0.001 ^ab^	0.024 ± 0.004 ^bc^
	ZnF+Bpa	1.23 ± 0.16 ^bc^	1.57 ± 0.06 ^ab^	1.67 ± 0.16 ^bc^	2.03 ± 0.08 ^b^	0.70 ± 0.09 ^bc^	0.60 ± 0.04 ^bc^	23.5 ± 2.1 ^b^	20.2 ± 2.4 ^bc^	0.039 ± 0.004 ^ab^	0.025 ± 0.006 ^bc^
	ZnS+Bpa	1.16 ± 0.12 ^bc^	1.41 ± 0.19 ^bc^	1.57 ± 0.15 ^c^	1.97 ± 0.25 ^bc^	0.63 ± 0.05 ^bc^	0.50 ± 0.08 ^bc^	24.4 ± 1.9 ^b^	16.3 ± 0.8 ^cd^	0.032 ± 0.006 ^bc^	0.018 ± 0.002 ^c^
DS	C	0.72 ± 0.03 ^d^	1.55 ± 0.11 ^ab^	1.03 ± 0.03 ^d^	2.27 ± 0.19 ^ab^	0.33 ± 0.03 ^c^	0.57 ± 0.04 ^bc^	26.1 ± 1.3 ^ab^	21.0 ± 2.2 ^bc^	0.040 ± 0.002 ^ab^	0.049 ± 0.008 ^a^
	ZnP	1.23 ± 0.14 ^bc^	1.75 ± 0.06 ^ab^	1.60 ± 0.17 ^bc^	2.37 ± 0.11 ^ab^	0.73 ± 0.10 ^b^	0.57 ± 0.07 ^bc^	19.2 ± 1.9 ^cd^	20.9 ± 3.8 ^bc^	0.043 ± 0.003 ^ab^	0.042 ± 0.004 ^ab^
	ZnF	1.38 ± 0.11 ^bc^	1.51 ± 0.12 ^b^	1.83 ± 0.15 ^bc^	2.03 ± 0.15 ^b^	0.90 ± 0.03 ^ab^	0.50 ± 0.02 ^bc^	31.4 ± 0.8 ^a^	20.7 ± 4.3 ^bc^	0.035 ± 0.005 ^bc^	0.032 ± 0.006 ^bc^
	ZnS	1.06 ± 0.20 ^cd^	1.76 ± 0.12 ^ab^	1.53 ± 0.21 ^cd^	2.33 ± 0.19 ^ab^	0.47 ± 0.09 ^c^	0.50 ± 0.03 ^bc^	26.5 ± 1.9 ^ab^	19.9 ± 2.8 ^c^	0.036 ± 0.002 ^bc^	0.032 ± 0.004 ^bc^
	Bpf	1.19 ± 0.13 ^bc^	1.03 ± 0.07 ^cd^	1.63 ± 0.17 ^bc^	1.47 ± 0.05 ^cd^	0.63 ± 0.13 ^bc^	0.37 ± 0.05 ^c^	28.4 ± 1.3 ^a^	16.5 ± 1.8 ^cd^	0.045 ± 0.001 ^ab^	0.047 ± 0.006 ^ab^
	ZnP+Bpf	1.03 ± 0.18 ^cd^	1.67 ± 0.03 ^ab^	1.47 ± 0.23 ^cd^	2.27 ± 0.06 ^ab^	0.53 ± 0.12 ^bc^	0.60 ± 0.10 ^bc^	21.6 ± 1.7 ^bc^	24.3 ± 2.5 ^b^	0.045 ± 0.003 ^ab^	0.027 ± 0.005 ^bc^
	ZnF+Bpf	1.31 ± 0.20 ^bc^	1.09 ± 0.14 ^c^	1.73 ± 0.26 ^bc^	1.53 ± 0.19 ^cd^	0.87 ± 0.08 ^ab^	0.40 ± 0.03 ^c^	26.3 ± 1.3 ^ab^	15.4 ± 1.5 ^cd^	0.022 ± 0.002 ^c^	0.028 ± 0.007 ^bc^
	ZnS+Bpf	0.75 ± 0.06 ^cd^	1.17 ± 0.05 ^bc^	1.10 ± 0.11 ^d^	1.60 ± 0.09 ^bc^	0.33 ± 0.02 ^c^	0.43 ± 0.01 ^c^	13.8 ± 1.4 ^d^	15.7 ± 3.0 ^cd^	0.039 ± 0.005 ^ab^	0.044 ± 0.002 ^ab^
	Bpa	0.95 ± 0.11 ^cd^	1.36 ± 0.07 ^bc^	1.33 ± 0.20 ^cd^	1.87 ± 0.06 ^bc^	0.43 ± 0.02 ^c^	0.50 ± 0.08 ^bc^	25.4 ± 0.7 ^b^	15.2 ± 0.6 ^cd^	0.032 ± 0.003 ^bc^	0.037 ± 0.004 ^b^
	ZnP+Bpa	1.00 ± 0.03 ^cd^	1.76 ± 0.11 ^ab^	1.43 ± 0.01 ^cd^	2.30 ± 0.14 ^ab^	0.57 ± 0.06 ^bc^	0.57 ± 0.03 ^bc^	18.8 ± 2.4 ^cd^	14.8 ± 2.6 ^cd^	0.026 ± 0.006 ^bc^	0.046 ± 0.006 ^ab^
	ZnF+Bpa	1.35 ± 0.13 ^bc^	1.10 ± 0.19 ^c^	1.67 ± 0.16 ^bc^	1.47 ± 0.22 ^cd^	0.57 ± 0.07 ^bc^	0.40 ± 0.09 ^c^	26.0 ± 0.6 ^ab^	18.3 ± 4.0 ^cd^	0.027 ± 0.005 ^bc^	0.033 ± 0.009 ^bc^
	ZnS+Bpa	1.38 ± 0.10 ^bc^	1.35 ± 0.12 ^bc^	1.90 ± 0.11 ^bc^	2.00 ± 0.18 ^bc^	0.73 ± 0.03 ^b^	0.47 ± 0.14 ^bc^	20.9 ± 1.7 ^bc^	20.1 ± 0.8 ^bc^	0.028 ± 0.002 ^bc^	0.027 ± 0.006 ^bc^

^1^ NS (no stress, 100% irrigation), DS (drought stress, 60% irrigation), C (control), ZnP (zinc priming), ZnF (foliar zinc), ZnS (soil zinc), Bpf (*P. fluorescens*), Bpa (*P. aeruginosa*). Values are expressed as mean ± SE (n = 3). Different lowercase letters indicate significant differences (*p* < 0.05, LSD test) between treatment means within each column (i.e., for each individual trait × year combinations).

**Table 3:** Effect of year on some physiological and morphological traits of maize.

Year	Chlorophyll *b* (mg·g^−1^ FW)	LAI	Catalase Activity (mmol·g^−1^ FW·min^−1^)
2018	0.39 ± 0.0120 ^b1^	3.84 ± 0.056 ^b^	183.10 ± 5.3 ^a^
2019	0.50 ± 0.0124 ^a^	4.06 ± 0.059 ^a^	112.33 ± 5.0 ^b^

^1^ values are expressed as mean ± SE (n = 72). Different lowercase letters indicate significant differences (*p* < 0.05, LSD test) in each column.

**Table 4:** The effect of drought stress on some physiological traits of maize.

Treatment	Chlorophyll *b* (mg·g^−1^ FW)	Glycine Betaine (µg·g^−1^ DW)	MDA (µmol·g^−1^ FW)	EL (%)	LAI
NS	0.42 ± 0.012 ^b1^	1.60 ± 0.043 ^b^	7.01 ± 0.057 ^b^	35.48 ± 1.0 ^b^	4.16 ± 0.06 ^a^
DS	0.46 ± 0.015 ^a^	1.98 ± 0.048 ^a^	8.34 ± 0058 ^a^	50.53 ± 1.2 ^a^	3.74 ± 0.05 ^b^

^1^ NS (no stress, 100% irrigation), DS (drought stress, 60% irrigation). Values are expressed as mean ± SE (n = 72). Different lowercase letters indicate significant differences (*p* < 0.05, LSD test) in each column.

**Table 5:** Impact of interaction between drought stress and combination of zinc sulfate and solvent-forming bacteria on soluble sugars and catalase of maize.

Treatment		Soluble Sugars (mg·g^−1^ FW)	Catalase Activity (mmol·g^−1^ FW·min^−1^)
NS	C	65.7 ± 3.9 ^bc1^	106.3 ± 9.3 ^b^
	ZnP	62.7 ± 4.2 ^bc^	154.0 ± 25.5 ^ab^
	ZnF	44.8 ± 5.0 ^cd^	110.8 ± 25.4 ^b^
	ZnS	47.0 ± 4.9 ^cd^	107.3 ± 17.2 ^b^
	Bpf	64.3 ± 2.8 ^bc^	156.2 ± 31.2 ^ab^
	ZnP+Bpf	53.8 ± 4.3 ^c^	187.0 ± 32.4 ^a^
	ZnF+Bpf	54.3 ± 2.0 ^bc^	124.5 ± 19.7 ^b^
	ZnS+Bpf	67.7 ± 4.8 ^b^	128.5 ± 20.9 ^b^
	Bpa	34.0 ± 2.6 ^d^	125.3 ± 17.2 ^b^
	ZnP+Bpa	32.3 ± 1.7 ^d^	156.3 ± 28.5 ^ab^
	ZnF+Bpa	49.5 ± 5.9 ^ab^	106.5 ± 9.9 ^b^
	ZnS+Bpa	60.2 ± 4.7 ^bc^	133.5 ± 19.2 ^b^
DS	C	43.0 ± 4.8 ^cd^	163.3 ± 21.4 ^ab^
	ZnP	61.2 ± 2.1 ^bc^	174.7 ± 21.0 ^ab^
	ZnF	37.7 ± 3.1 ^d^	126.0 ± 11.8 ^b^
	ZnS	55.2 ± 2.4 ^bc^	180.3 ± 23.3 ^ab^
	Bpf	63.7 ± 4.6 ^bc^	179.3 ± 25.6 ^b^
	ZnP+Bpf	81.5 ± 5.3 ^a^	202.3 ± 20.1 ^a^
	ZnF+Bpf	59.5 ± 9.3 ^bc^	132.0 ± 20.6 ^b^
	ZnS+Bpf	65.8 ± 10.4 ^bc^	168.7 ± 15.6 ^ab^
	Bpa	40.8 ± 2.8 ^cd^	175.5 ± 18.1 ^b^
	ZnP+Bpa	53.7 ± 5.5 ^c^	137.0 ± 16.3 ^b^
	ZnF+Bpa	54.8 ± 7.2 ^bc^	128.8 ± 13.1 ^b^
	ZnS+Bpa	63.2 ± 8.4 ^bc^	184.8 ± 30.6 ^ab^

^1^ NS (no stress, 100% irrigation), DS (drought stress, 60% irrigation), C (control), ZnP (zinc priming), ZnF (foliar zinc), ZnS (soil zinc), Bpf (*P. fluorescens*), Bpa (*P. aeruginosa*). Values are expressed as mean ± SE (n = 6). Different lowercase letters indicate significant differences (*p* < 0.05, LSD test) in each trait. Data derived from significant two-way interactions (drought × treatment) following ANOVA in Table 1 and Table 6.

**Table 6:** Combined analysis of variance (mean squares) for physiological and antioxidant enzyme traits of maize under drought stress: peroxidase activity (POD, unit·min^−1^), superoxide dismutase activity (SOD, μmol·min^−1^), catalase activity (CAT, μmol·g^−1^ FW·min^−1^), leaf area index (LAI), and electrolyte leakage (EL, %).

S.O.V.	D.F.	Mean Squares				
		EL	LAI	CAT	SOD	POD
Year (Y)	1	36.40 ^ns^	1.72 *	180,271.01 **	0.1805 **	0.000136 ^ns^
Replicate (R) × Y	4	37.85	0.1	1477.78	0.0027	0.000085
Drought stress (D)	1	8157.10 **	6.34 *	7267.56 *	0.1331 ^ns^	0.004096 **
D × Y	1	24.17 ^ns^	0.24 ^ns^	7070.01 ^ns^	0.0016 **	0.00060 ^ns^
R × Y × D	4	202.3	0.36	58.22	0.0039	0.000033
Zn—Bacteria (ZB)	11	96.21 ^ns^	0.36 *	5807.58 **	0.0021 ^ns^	0.000254 **
Y × ZB	11	81.56 ^ns^	0.34 ^ns^	1550.07 ^ns^	0.0015 ^ns^	0.000059 ^ns^
D × ZB	11	63.40 ^ns^	0.053 ^ns^	4262.38 *	0.0011 ^ns^	0.000251 **
Y × D× ZB	11	50.30 ^ns^	0.130 ^ns^	2257.85 ^ns^	0.0010 ^ns^	0.000136 *
Error	88	103.02	0.178	1799.07	0.0027	0.000052
C.V. (%)		23.6	10.68	18.71	23.11	23.74

*, ** and ^ns^: significant at 5, 1% of probability and non-significant, respectively.

## Data Availability

Data will be made available on request.

## Abbreviations

The following abbreviations are used in this manuscript:

LAI	leaf area index
PCA	principal component analysis
Zn	zinc
PGPR	plant-growth-promoting rhizobacteria
MDA	malondialdehyde
SOD	superoxide dismutase
CAT	catalase
POD	peroxidase
EL	electrolyte leakage
